# Iodine Deficiency in Zhejiang Pregnant Women in the Context of Universal Salt Iodization Programme

**DOI:** 10.1038/s41598-018-26942-z

**Published:** 2018-06-11

**Authors:** Zhifang Wang, Mingluan Xing, Wenming Zhu, Guangming Mao, Zhe Mo, Yuanyang Wang, Zhijian Chen, Xiaoming Lou, Shichang Xia, Xiaofeng Wang

**Affiliations:** 1 Zhejiang Provincial Center for Disease Control and Prevention, Department of Environmental and Occupational Health, Hangzhou, 310051 China; 2 Zhejiang Provincial Center for Disease Control and Prevention, Key Medical Research Center, Hangzhou, 310051 China

## Abstract

Zhejiang introduced universal salt iodization (USI) programme in 1995 and has achieved the goal of elimination of iodine deficiency disorders (IDD) since 2011. However, no systematical data of iodine nutritional status in population in pregnancy is available. In this cross-sectional study, pregnant women were interviewed to complete questionnaires in addition to handing in samples of urine and household table salt between March 2016 to February 2017. Date of birth, age of pregnancy, ethnicity and dietary iodine habits were recorded. The overall median urinary iodine concentration in 8561 pregnant women was 130.47 µg/L, which was lower than the cut-off value of iodine sufficiency of 150 µg/L recommended by the WHO. Participants using non-iodized salt, taking non-iodine-containing supplements, in coastal and in Han group were independently associated with iodine deficiency. The current USI programme did not supply Zhejiang pregnant women with sufficient iodine intake. They are generally iodine deficient, which have great public health importance since even mild IDD in pregnancy have adverse effects on fetal neurodevelopment. We strongly recommend urgent measures to improve iodine intake in pregnancy.

## Introduction

Adequate iodine intake is important during all stages of life, as iodine is essential to produce thyroid hormones to the normal metabolic rate and brain development, especially during pregnancy. A lack of iodine in the diet in pregnancy may result in iodine deficiency disorders (IDD). IDD is the most common public health problem worldwide, with approximately 2 billion population affected^1^. The most devastating consequences of IDD include stillbirth, spontaneous abortion, mental retardation, congenital abnormalities, and endemic cretinism.

Iodine nutritional status during pregnancy can be assessed by measurement of urinary iodine concentration (UIC) in representative samples from the population of pregnant women, as recommended by the WHO^2^. The median UIC below 150 μg/L is considered as iodine deficiency and the value of 150–249 μg/L indicates sufficiency.

IDD is among the easiest of all nutrient disorders to prevent. Salt iodization programme is the main strategy to combat IDD. IDD was one of the most serious public health threat in China in the 1990s, with at least 8 million endemic cretinism cases^3,4^. China introduced universal salt iodization (USI) in 1995. After two decades’ mandatory use of iodized salt, a substantial progress has made and the goal of elimination of IDD has been achieved. Since then, the interventive measures of combating IDD has been taking account for preventing and controlling excessive iodine intake in population. Based on the China’s 2011 National IDD Surveillance results showing that iodine intake status in the general population were above requirement^5^, iodine content in household table salt therefore started to drop from 35 ppm during 2000–2011 to 25 ppm in 2012.

The introduction of the new decreased iodine content in household table salt may put population in pregnancy at high risk to IDD since they have a higher iodine requirement than the general population to maintain both maternal and fetal normal neurodevelopment. Previous IDD surveillances studies showed iodine deficiency in pregnant women emerged in the coastal provinces^6–9^ where household coverage of iodized salt was less than the lower limit of criteria of China’s Elimination of IDD (95%; GB 16006-2008). However, the number of pregnant women participating in these studies was limited, which could not build up the overall picture of iodine status in population in pregnancy. The objective of this present study was to assess systematically the iodine status of pregnant women using the standard approach of targeting to the most relevant geographical locations and dietary iodine resources.

## Results

### Demographic characteristics of the total participating pregnant women

A total of 9,196 pregnant woman were enrolled. Of them, 225 pregnant women (2.45%) self-reported a historical diagnosis of thyroid diseases or any disorders of thyroid function and 8,895 (96.73%) provided samples of urine and table salt. The final analysis was 8,561 participants (93.09%) who completed a face-to-face questionnaire in addition to handing in samples.

Table 1 shows the demographic characteristics in the 8561 participating pregnant women. Though 25 ethnic groups participated in this study, Han was the majority ethnicity since Han accounted for 96.39% (8,252) of the total of participants. The two main minority groups were She (0.71%, 61) and Hui (0.20%, 17), respectively. The mean age of the total participants was 29.30 years old (SD: 4.91; Range: 15.68–48.48 years). The average of gestational age was 20.72 weeks (SD: 9.04; Range: 1–42 weeks).

**Table 1:** UIC analysed by sociodemographic characteristics.

Sociodemographic characteristics	N (%)	Median UIC (μg/L)	*P*
**Age groups (Years old)**			
15.0–29.9	5167 (60.4)	130.91 (83.50–199.00)	0.652^*^, 0.668
30.0–49.9	3394 (39.6)	129.89 (81.53–200.00)	
**Trimester**			
1st	2391 (27.9)	139.00 (85.85–202.58)	0.018^*^, 0.023
2nd	3797 (44.4)	131.90 (82.37–201.85)	
3rd	2373 (27.7)	125.00 (79.81–191.23)	
**Ethnic groups**			
Han	8252 (96.4)	129.70 (82.20–198.47)	0.006^*^, 0.007
Others	309 (3.6)	151.94 (91.82–215.10)	
**Highest educational level**			
Middle school and below (≤9 years)	2997 (34.9)	132.48 (83.51–202.47)	0.171^*^, 0.080
High school (10–12 years)	2086 (24.4)	127.00 (81.73–199.20)	
College and above (≥13 years)	3478 (40.6)	130.49 (82.11–197.40)	
**Income**^**^**^ (**RMB)**			
≤29,999	1492 (17.4)	131.03 (84.50–203.13)	0.513^*^, 0.230
30,000–59,999	3381 (39.5)	132.27 (84.10–197.33)	
60,000–99,999	2351 (27.5)	128.34 (81.84–200.00)	
≥10,000	1337 (15.6)	128.00 (79.97–200.01)	
Total	8561 (100)	130.47 (82.30–199.97)	—

^*^Mann-Whitney tests were performed for comparison of two groups of non-normal data and Kruskal-Wallis tests were for more than two groups. Mantel-Haenszel test of linear association was performed to test for linear trend between variables. ^**^**^Household net income per capita in 2015.

### Overall median UIC in pregnant women

The frequency of UIC among the participating pregnant women was a positive skewness distribution (P < 0.001). The median UIC in total studying pregnant women was 130.47 µg/L (IQR: 82.30–199.97 µg/L). According to the WHO recommended level, 58.3% (4,990) of the participants belonged to iodine deficiency (<150 µg/L) and 27.1% (2,318) had iodine sufficiency (150–249 µg/L).

Among the sociodemographic characteristics, ethnicity was associated with the significant difference in the median UIC (P_Mantel-Haenszel-test_ = 0.007; Table 1). The median of UIC in Han group (129.70 μg/L) was significantly lower than that in minority groups (151.94 μg/L; P_Mann-Whitney-test_ = 0.006). Median UIC in pregnancy steadily decreased from the first trimester (139.00 µg/L) to the second (131.90 µg/L;) and then to the third (125.00 µg/L; P_Kruskal-Wallis-test_ = 0.018;). Samples in the third trimester was independently associated with the lowest UIC (P_Mantel-Haenszel-test_ = 0.023). No other sociodemographic factor was defined to be significantly associated with low UIC (P_age_ = 0.652, P_education_ = 0.171, and P_income_ = 0.513, respectively; Table 1).

In general, iodine status in Zhejiang pregnant women was distributed with regional difference (P_Kruskal-Wallis-test_ < 0.001), with the lowest median UIC recorded in the coastal region (119.38 µg/L), the medium in the sub-coastal (131.30 µg/L) and the highest in the inland (146.00 µg/L; P_Mantel-Haenszel-test_ < 0.001). Similarly, among those participants in each trimester, percentage of participants with iodine deficiency was gradually decreased from the coastal to the sub-coastal and then to the inland (Table 2).

**Table 2:** UIC analysed according to participating regions.

Trimester	Region	n	Median UIC (μg/L)	*P*
1st	Coastal	870	127.17 (79.93–193.07)	0.023^*^, 0.028
	Sub-coastal	1085	133.05 (89.35–199.41)	
	Inland	565	134.95 (82.99–217.50)	
2nd	Coastal	1684	119.74 (73.80–191.98)	0.000^*^, 0.000
	Sub-coastal	1406	131.23 (85.13–193.52)	
	Inland	1171	150.69 (91.32–218.58)	
3rd	Coastal	897	113.00 (75.32–173.69)	0.000^*^, 0.000
	Sub-coastal	646	130.82 (83.49–196.00)	
	Inland	571	142.00 (95.86–225.34)	
Total	Coastal	3310	119.38 (75.58–187.20)	0.000^*^, 0.000^*^
	Sub-coastal	3023	131.30 (85.66–196.00)	
	Inland	2228	146.00 (89.68–219.09)	

^*^Kruskal-Wallis tests were performed for three groups. Mantel-Haenszel test of linear association was performed to test for linear trend.

### Median UIC in pregnancy by dietary iodine habits

Samples in using non-iodized salt (P_Mantel-Haenszel-test_ < 0.001), non-iodine-containing supplements (P_Mantel-Haenszel-test_ < 0.001) and seafood (P_Mantel-Haenszel-test_ < 0.001) were independently associated with low UIC (Table 3). We performed a binary logistic regression analysis to investigate the associations between sociodemographic characteristics (ethnicity and trimester), geographical locations (coastal, sub-coastal, and inland), and dietary iodine habit (category of consumed salt, seafood consumption, iodine-containing supplements intake) and establish the probability of iodine deficiency in pregnancy (Table 4). Pregnant women consuming non-iodized salt had a 1.7 times higher probability of iodine deficiency than those consuming iodized salt (P < 0.001). Pregnant women taking non-iodine-containing supplements had a 1.8 times higher probability than those taking iodine-containing supplements (P < 0.001). The probability of iodine deficiency in those living in coastal and in subcoastal were 1.5 times (P < 0.001) and 1.3 times (P < 0.001), respectively, higher than those in land. Pregnant women in Han had 1.4 times higher probability of iodine deficiency than those in minority groups (P < 0.001). No independently significant difference was defined between trimester of pregnancy (P = 0.247), seafood consumption (P = 0.344) and iodine deficiency.

**Table 3:** UIC analyzed according to dietary iodine habits.

Dietary iodine habits	Number (%)	Median UIC (IQR), µg/L	*P*
**Often consume iodized salt**			0.000^*^, 0.000
Yes	7716 (90.1)	134.41 (84.81–202.57)	
No	845 (9.9)	102.50 (62.57–159.03)	
**Often consume seafood**			0.000*, 0.000
Yes	2011 (23.5)	123.00 (75.80–188.40)	
No	6550 (76.5)	132.50 (84.59–202.15)	
**Often take iodine-containing supplements**			0.000^*^, 0.000
Yes	465 (5.4)	161.10 (107.05–229.55)	
No	8096 (94.6)	128.58 (81.50–197.76)	

^*^Mann-Whitney tests were performed for comparison of two groups of non-normal data. Mantel-Haenszel test of linear association was performed to test for linear trend.

**Table 4:** UIC analyzed according to binary logistic regression model.

Influential factors	B	SE	*P*	OR (95% CI)
Ethnicity (Han = 1, others = 0)	0.318	0.081	0.000	1.374 (1.172–1.610)
Region (coastal = 1, inland = 0)	0.436	0.041	0.000	1.547 (1.427–1.677)
Region (sub-coastal = 1, inland = 0)	0.276	0.040	0.000	1.318 (1.219–1.425)
Trimester	0.038	0.022	0.247	1.035 (0.976–1.097)
Iodized salt (Yes = 0, No = 1)	0.541	0.056	0.000	1.718 (1.538–1.919)
Seafood (Yes = 1, No = 0)	0.037	0.040	0.344	1.038 (0.961–1.122)
Iodine-containing supplements (Yes = 1, No = 0)	−0.580	0.056	0.000	0.552 (0.456–0.667)

SE: Standard Error.

Since the highest proportions of participants with iodine deficiency were recorded in the coastal, we compared the dietary iodine habits between regions. Of these 3 regions, the coastal participants had the significantly lower proportion of consumption of iodized salt (81.81%, 2,708; P = 0.003), when compared with that in the subcoastal (95.40%, 2,884) and the inland (95.33%, 2,124). In addition, the coastal participants had the highest proportion in consuming seafood (42.00%, 1,391; P < 0.001), while the sub-coastal and the inland had 13.60% (410) and 9.40% (210), respectively. There was no significant difference for supplementing containing iodine intake between regions (P = 0.33).

Regarding dietary habits between ethnicities, pregnant women in Han group had a significantly higher percentage of consuming non-iodized salt (10.1%, 836) than those in minority groups (2.9%, 9; P < 0.001) while those using seafood and iodine supplements were not of differences.

We then established the effects of dietary iodine habits (seafood, iodized salt and iodine-containing supplements), and geographical locations (coastal, sub-coastal, and inland) on iodine deficiency via a generalized linear model assuming a binary logistic distribution, based on the minimum value of Akaike’s Information Criterion (AIC). Participants who consumed non-iodized salt (P < 0.001), took no iodine-containing supplements (P < 0.001) and were from the coastal region (P < 0.001) were all independently associated with iodine deficiency. There was no independent association with seafood (P = 0.275). Further detailed analysis showed that there were significantly interactions between iodized salt, iodine-containing supplements and geographical locations on iodine deficiency (P < 0.001). For those only consuming iodized salt, participants in these 3 regions were all iodine deficient, with lower median UICs of 122.26 µg/L (2,574) in coastal, 131.00 µg/L (2,704) in sub-coastal and 147.30 µg/L (2,031) in inland, respectively. However, those participants from each region using both iodized salt and iodine-containing supplements were sufficient, with the median UICs of 158.53 µg/L (134) in coastal, 163.99 µg/L (180) in sub-coastal and 173.00 µg/L in inland, respectively.

## Discussion

This is the first large-scale observational survey on iodine nutritional status covering 8561 pregnant women across 3 regions (coastal, sub-coastal and inland) in Zhejiang province, after USI program has been implemented for two decades. Zhejiang has achieved the goal of elimination of IDD since 2010^10^. However, our study showed that Zhejiang population in pregnancy was generally iodine deficient (median UIC 130.47 µg/L), which have great public health importance since even mild IDD in pregnancy have adverse effects on fetal neurodevelopment. Our finding in this study is consistent with a previous study conducted in the remaining 8 environment-lacking-iodine regions including Tianjin municipality, Hebei province, Shanghai municipality, Fujian province, Shandong province, Guangdong province, Guangxi Zhuang Autonomous Region, and Hainan province^10^. In fact, pregnant women are widespread iodine deficient in the developing and developed countries around the world^11–30^. European pregnant women in many countries, including Belgium^11^, the Czech Republic^12^, Spain^13,14^, the United Kingdom^15–17^, Denmark^18^, Norway^19^, and France^20,21^, and those in the United States^20,22^, Australia^23^ and New Zealand^24^ have been documented well inadequate iodine intake. Nearly 80% of pregnant women in Vietnam^25^, 70% in Philippine^26^ and more than half in Pakistan^27^ had low UIC, while in Africa^28,29^ and in south America^30^, pregnant women have already recorded iodine deficiency. Our findings confirm this information and further emphasize the need to surveillance pregnant women in intervals, even after the goal of elimination of IDD have achieved.

Our study showed that iodine deficiency in Zhejiang pregnant women with regional difference. Mild iodine deficiency appeared in the coastal and the sub-coastal and borderline iodine sufficiency in the inland. This regional difference may be related with different household coverage of iodized salt between regions. Coastal households have an easier access to sea salt, without adding iodine, which was produced from salt evaporation ponds and freely distributed in market, caused by the relaxed salt monopoly after elimination of IDD has achieved. At the beginning of introduction of universal salt iodine programme, China made it mandatory through legislation^31^ and required the salt industry must sell only iodized salt on the market’s shelf to the population in iodine deficient areas and make non-iodized salt only available on showing a doctor’s prescription in specified places. However, non-iodized salt became readily available on the market after elimination of IDD achieved. Another potential factor related with incidence of thyroid disease may have an adverse effect on the use of iodized salt in the coastal region. With the mature of salt iodization program and the risk of excessive iodine intake overestimated, the incidence of thyroid cancer is reported on the rising in the coastal regions^32–34^ and population began to blame the use of iodized salt on the increasing thyroid cancer incidence. Consequently, dietary habits of iodized salt silently change. The trend of household coverage of iodized salt in coastal residents appeared downward, decreasing from more than 95% during 1997–2002^35^ to 90–95% in 2011^36^ and to nearly 80% during 2014^14^. This decreasing household coverage of iodized salt presented a substantial challenge to combat IDD, as recently addressed in Vietnam^37^. The number of household using iodized salt in Vietnam halved after the official declared achievement of elimination of IDD and falling iodine level in pregnant women was also observed.

Trimester was found to be progressively associated with low UIC, with the lowest recorded in the third trimester and the highest in the first. This result is consistent with the studies in Switzerland^38^ and Iran where pregnant women are iodine sufficiency^39^. Consumption of iodized salt in trimester gradually decreased, caused by edema in pregnancy, may contribute to a low UIC. However, the reasons of the continuous decrease of the median UIC throughout pregnancy are complex. Further studies are needed to elucidate this problem.

Pregnant women in Han group had the lower UIC than those in minority groups. Since iodized salt remains the main dietary iodine source, unsurprisingly, we found the percentage of use of iodized salt in Han pregnant women was lower than that in other groups. Furthermore, studies on the association between ethnicity and iodine deficiency are still scarce. Whether genetic diversity exists in UIC during pregnancy remains unknown.

IDD in population residing in the areas where natural sources of iodine are too low to maintain optimal iodine intake status will persist until iodine enters their food, e.g. iodized salt, iodized cows’ milk and bread, drinking water, seafood, and iodine-containing supplements. Iodized milk and bread is rarely available to purchase in China. Though the researches related to iodine content in drinking water and soil across China are not available, one study has shown drinking water, soil and the food products planted in these areas is generally lacking iodine in the coastal regions of China^40^. Our study showed that pregnant women still were iodine deficient even when coverage rate of iodized salt at a household level reached a substantial high level of 95%. In the current context of USI program, two main public health measures related with iodized salt may contribute to inadequate dietary iodine intake in pregnancy. First, the iodine content in salt has been decreased since 2012. As expected, urinary iodine excretion in school-age students decreased from 238.6 μg/L in 2011^5^ to 179.0 μg/L in 2014^14^, representing previous iodine status of above requirement in the general population evolving into the optimal iodine level (100–199 μg/L). Instantaneously, decreased UIC is emerging in pregnant women^14,41^, as shown above in this study. Second, to reduce the risk of cardiovascular diseases, dietary salt intake has been reduced from 12 g/day/person in 2007^42^ to 8 g/day/person during 2009–2012 in this area^43^. Indeed, WHO recommends reducing the salt intake to 5 g/day/person^44^. Therefore, a salt intake below 5 g/day in pregnancy may cause iodine deficiency and more urgent interventive measures should be taken to ensure adequate iodine intake in pregnant women^45^.

Though WHO recommends that universal salt iodization program remains the main strategy for elimination of IDD in pregnancy, iodine-containing multivitamin supplements have been suggested to take in pregnant women and documented well to improve the UIC in pregnancy in many countries, including Australia^23,46^, the United States^47^, and many European countries^48^, Currently, there is no official suggestion for iodine-deficient pregnant women to take iodine supplements. Our study showed that only 5% of pregnant women took iodine supplements. This figure was less than that in countries where the official governments have a suggestion, for example, 70% of Swiss women^48^, nearly 50% of Hungarian pregnant women^48^ and 30% of Danish pregnant women^48^ received supplements containing iodine. Our study showed that iodized salt and iodine supplements, but not seafood, were the main dietary iodine sources.

In the context the adoption of the new decreased iodine content in salt, the goal of USI programme is to perfectly achieve iodine sufficiency in pregnant women without inducing excessive iodine intake in the general population. Epidemiological studies have shown the iodine nutritional status in school age students have been iodine sufficient^14^. There is population-to-population variation in consumption of iodized salt. Because children attending the primary school have been supplemented by the Ministry of Health with iodine, vitamin D, protein, and iron preparations via school meal programme even though they don’t consume iodized salt at their households. It appears impractical to use two different iodine contents in salt at a household level to achieve optimal iodine intake in different populations, with 25 ppm in the general population and 30 ppm in pregnant women. Taken together, we believe that iodine fortification of Zhejiang salt cannot supply pregnant women with sufficient iodine. In addition to implementation of USI, iodine supplementation should be recommended by the healthcare providers, including obstetricians and gynecologists, since they have been proved to play a critical role in increasing knowledge and awareness of the beneficial effects of iodine supplementation during pregnancy^49^.

There is one limitation in this study. We adopted the median UIC of 150–249 μg/L to assess adequate iodine status among pregnant women recommended by the WHO. The lower cut-off value of 150 μg/L was established based on the mean daily urinary volume of 1.5 L. However, an increase of glomerular filtration rate occurs during pregnancy, which induce an increased daily urinary volume and finally lead to an overestimation iodine deficiency status in pregnancy. To eliminate this potential bias affecting iodine nutrition in population, the ratio of UIC to creatinine will be suggested in future studies^50^.

## Conclusions

We believe that our findings have great public health importance for Zhejiang province and the other coastal regions of China in the context of USI programme. Iodine deficiency in pregnancy presents a substantial challenge to a sustainable elimination of IDD. This study confirmed that pregnant women in this area had inadequate iodine intake when only consuming iodized salt while they may have adequate intake when using both iodized salt and iodine supplements. A comprehensive investigation of iodine status in pregnant women across China is urgently needed and evidence-based recommendations on ensuring adequate iodine status in pregnancy are also needed.

## Methods

### Sampling and participants

This was a cross-sectional study embedded into the 2016 China’s National IDD Surveillance Programme. Participants were recruited from 3 regions (coastal, sub-coastal and inland) across Zhejiang province, an eastern part of China. Sampling was in accordance with the WHO’s and China’s National IDD Surveillance guidelines^51^. The sampling method was detailed in our previous study^9^. Briefly, the total of 89 county-level divisions were all selected. For each selected county, five towns were randomly selected from five different geographical locations (east, west, south, north and center). For each selected town, total of 20 pregnant women routinely visiting antenatal care clinics were invited to participate this study. Only those participants residing in the selected town for at least 6 months were qualified to enrolled in this study.

### Procedures

A 10 mL random spot urine sample and a 30 g household table salt sample were obtained from each participant between March 2016 to February 2017. UIC was determined by using the colorimetric ceric ion arsenious acid method. Iodine content in salt was measured using titrimetric method with sodium thiosulphate. All the iodine laboratories participated in an interior quality control and an external quality assurance programme run by the National Center for Disease Control and Prevention of China.

Each enrolled participant was interviewed to fill in the questionnaires by the trained investigators. Date of birth, ethnicity, occupation, age of pregnancy, education, household net income per capita in 2015, history of thyroid disease, history of thyroid dysfunction, and frequency of dietary iodine sources (seafood, iodized salt and iodine-containing supplements) per week were recorded. Seafood includes sea vegetables (like kelp, Kombu, Wakame, Nori, and Hijiki) and sea fish, shrimp, lobster, and scallop. Iodized salt is classified as the salt containing at least 5 ppm iodine.

### Statistical analysis

SPSS software (version 23; Chicago, IL, USA) was used for all analyses. The Kolmogorov-Smirnov test was used to test for normality. Maternal age and gestational age were expressed as mean and standard deviation (SD). UIC was expressed as median and interquartile range (IQR). Mantel-Haenszel test of linear association was performed to test for linear trend of the ordinal variables, e.g. between trimesters, geographical locations and iodine deficiency. Mann-Whitney tests were performed for comparison of two groups of non-normal data and Kruskal-Wallis tests were for more than two groups. A binary logistic regression model was used to demonstrate the potential influential factors (sociodemographic characteristics, geographical locations, and dietary iodine habits) associated with the probability of iodine deficiency. A generalized linear logistic regression model was used to explore the multivariable effects of dietary habits of iodine intake and geographical locations on iodine deficiency. Further analyses of all two-factor interactions were considered. All statistical tests were two-tailed and analyses where *P* value is 0.05 and below were determined to be statistically significant.

For the final purpose of statistical analyses, frequency of consumption iodized salt, seafood and iodine-containing supplements were categorized into: (i) yes if the frequency of consumed food last week is more than 4 times; and (ii) no if less than 4 times last week. Maternal age was analyzed as a categorical variable: 15–29 years and 30–49 years. All the data involved in this study would be available as request.

### Ethical approval and informed consent

All research protocols involved in this study were approved by the Ethics Committee of Zhejiang Provincial Center for Disease Control and Prevention (ZJ-20160421). All methods were carried out in accordance with the relative guidelines and regulations. Written informed consents were obtained from all participants prior to enrolment.

### Data availability

All datasets generated or analysed during the current study are available from the corresponding authors on reasonable request.
